# BRCA1-BARD1: the importance of being in shape

**DOI:** 10.1080/23723556.2019.1656500

**Published:** 2019-09-11

**Authors:** Manuel Daza-Martin, Ruth M. Densham, Joanna R. Morris

**Affiliations:** Birmingham Centre for Genome Biology and Institute of Cancer and Genomics Sciences, University of Birmingham, Birmingham, UK

**Keywords:** BRCA1, BARD1, PIN1, fork protection, isomerization, RAD51

## Abstract

The breast cancer type-1 susceptibility protein (BRCA1) contributes to genome integrity through homologous recombinational DNA repair and by protecting stalled replication forks from nucleolytic degradation. We recently discovered that fork protection requires a conformational change of BRCA1 unimportant to homologous recombination repair, indicating separate roles for BRCA1 in these pathways.

## Authors view

The breast cancer type-1 susceptibility protein (BRCA1) has a well-established role in directing the repair of DNA double strand breaks through homologous recombination (HR). BRCA1 both promotes resection and contributes to the association of the partner and localizer of BRCA2 (PALB2) with the breast cancer type-2 susceptibility protein (BRCA2) to sites of DNA damage, where the PALB2-BRCA2 complex acts to exchange the single stranded DNA (ssDNA) binding replication protein A complex (RPA) for the DNA repair protein RAD51 homolog 1. This exchange generates a RAD51-nucleofilament which subsequently performs strand invasion and synapse formation required for gene conversion. It is now also clear that when replication forks stall, BRCA1 plays a role in the protection of newly synthesized DNA from excessive resection: a process that also requires key HR factors including BRCA2, Fanconi Anemia complementation group D2 (FANCD2), and most significantly RAD51 (reviewed in^1^). Replication may stall at roadblocks or collisions formed between the transcription and replication machinery; at difficult to replicate regions, such as repetitive sequences or secondary DNA structures; or due to nucleotide shortages. While nuclease activity is required at reversed forks to resume DNA replication, uncontrolled resection is correlated with genome instability. However, nascent strand loss mediated by Meiotic recombination 11 homolog 1 (MRE11) and DNA replication ATP-dependent helicase/nuclease DNA2 can be prevented by loading RAD51 at exposed ssDNA.

Given that many of the proteins required to promote RAD51:RPA exchange in HR are also required for limiting the degradation of stalled replication forks one might assume that the role of BRCA1 in both processes would be related. However, our recent work found that while both BRCA1 and PALB2 are required to protect nascent DNA following hydroxyurea-induced replication fork stalling, their canonical interaction, important in HR, is dispensable.^2^ PALB2 can be recruited through RING finger protein 168 (RNF168) or through phosphorylated RPA^3,4^ and we speculate that these mechanisms, rather than recruitment through BRCA1, may predominate at stalled replication forks.

**Figure 1.**
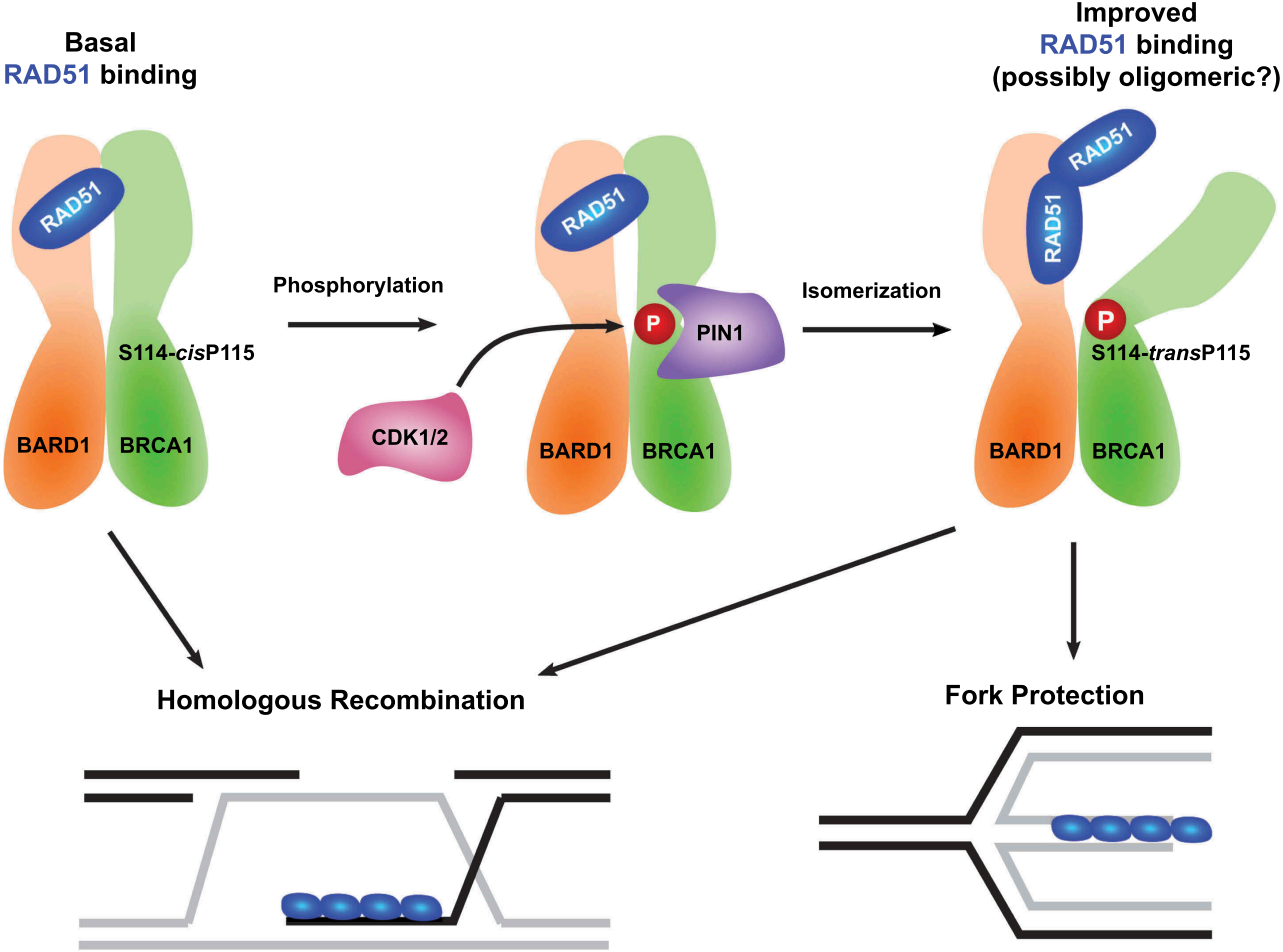
Phosphorylation and isomerization of BRCA1 promotes replication fork protection. Illustration of the post-translational modifications that regulate the heterodimeric breast cancer type-1 susceptibility protein (BRCA1) (green) and BRCA1-associated RING domain protein 1 (BARD1) (orange) complex. Cyclin-dependent kinases 1/2 (CDK1/2) (pink) phosphorylate BRCA1 at serine 114 which is bound by the peptidyl-prolyl *cis-trans* isomerase NIMA-interacting 1 (PIN1) (purple) to promote isomerization and improve the interaction with DNA repair protein RAD51 homolog 1 (RAD51) (blue) required for replication fork protection.

We found that the role of BRCA1 in fork protection relates to direct RAD51 interaction which is regulated through a series of post-translational events (Figure 1). We identified that phosphorylation of BRCA1 at serine 114 (S114) is required for fork protection but has no measurable impact on fork stalling, fork restart or features of HR.^2^ Phosphorylation of S114-BRCA1 requires cyclin-dependent kinases 1/2 (CDK1/2) and the phosphorylated BRCA1 region forms the main binding site for the phosphorylation-targeted peptidyl-prolyl *cis-trans* isomerase NIMA-interacting 1 (PIN1). PIN1 catalyzes the c*is-trans* isomerization of peptidyl bonds between phosphorylated serine/threonine and proline residues, resulting in subtle or significant conformational alterations of its substrates. In the case of BRCA1 and its heterodimeric binding partner BRCA1-associated RING domain protein 1 (BARD1), PIN1 acts to increase the accessibility of the RAD51 binding pocket on BARD1 (identified recently^5^), enhancing the ability of the heterodimer to bind RAD51.

Cells expressing a C-terminal BRCA2 mutation that inhibits fork protection, but that has no defect in HR, show genomic instability.^6^ Likewise we noted that cells with mutation of BRCA1-S114 that exhibit poor fork protection, but adequate HR, showed increased chromosomal breaks, which we imagine arise from the processing of stalled, de-protected structures.

The BARD1-RAD51 binding face on RAD51 overlaps with that of the BRCA2-RAD51 interaction face^5^ suggesting either that the total RAD51 needed at stalled forks interacts with BRCA1 and BRCA2 independently or that there is a hand-off between BRCA1 and BRCA2 reflecting different temporal needs during the process. Loss of chromodomain-helicase-DNA-binding protein 4 (CHD4) rescues fork protection defects in BRCA2-deficient cells yet does not restore protection to cells lacking BRCA1,^7^ while loss of RB binding protein-8/CtBP-interacting protein (RBBP8 commonly known as CtIP), which is epistatic with BRCA2 loss, further worsens fork protection in BRCA1-deficient cells.^8^ These observations are consistent with an ‘independent RAD51 populations’ model. However as yet there have been no reports of factor losses that rescue the need for BRCA1 but not BRCA2 in fork protection, perhaps suggesting a hierarchy, BRCA1> BRCA2, consistent with hand-off. The BRCA2 C-terminus binds and stabilizes oligomeric RAD51 and we speculate that the conformational change in BRCA1 may allow BRCA1-BARD1 to contribute a similar function.

CDK activity and PIN1 regulation are not sufficient to explain the observed recruitment of pS114-BRCA1 to stalled forks. It seems likely that the recently described interaction of BARD1 with newly made chromatin^9^ and BARD1 interaction with poly-ADP-ribose (PAR),^10^ present at stalled forks, contributes to heterodimer recruitment.

While RAD51-mediated preservation of nascent DNA at stalled forks is important to prevent chromosome damage, increased RAD51 at replication forks results in fork collapse and increased DNA breaks in unperturbed replication. We found that constitutive expression of a *trans*-favored form of BRCA1 similarly accelerated accumulation of DNA damage, suggesting that constitutive presentation of BRCA1-BARD1 primed for RAD51 interaction is dangerous. Murine Brca1 has a cysteine at the position equivalent to the PIN1 targeted proline of human BRCA1 so that the murine protein is likely to be constitutively present in a *trans*-favored form. Whether murine cells are more tolerant to Rad51 at replication forks or have another regulatory pathway remains a significant question.

Intriguingly, the regions surrounding the RAD51 binding region of BARD1 and the phosphorylation and PIN1 interaction region of BRCA1 contain a number of somatic and germline genetic cancer associated variants, some of which exhibit deficient fork protection without showing features of an HR-deficit. Whether impaired fork protection is enough to drive cancer development is not yet clear, but appears doubtful in view of a recent analysis of a murine *Bard1*-C-terminal mutant that exhibits poor fork protection, but shows no increased cancer development.^10^ Restoration of fork protection has recently emerged as means of resistance to HR-directed therapies in *BRCA1/2*-mutant cancers^1^ so that resolving the order and dependencies of BRCA1:BRCA2 and other critical ‘HR’ proteins in this pathway will be important to patient care.

## Figures and Tables

**Table:** 

BARD1	BRCA1-associated RING domain protein 1
BRCA1	breast cancer type-1 susceptibility protein
BRCA2	breast cancer type-2 susceptibility protein
CDK1/2	cyclin-dependent kinases 1/2
CHD4	chromodomain-helicase-DNA-binding protein 4
CtIP	CtBP-interacting protein
DNA2	DNA replication ATP-dependent helicase/nuclease
FANCD2	Fanconi Anemia complementation group D2
HR	homologous recombination
MRE11	Meiotic recombination 11 homolog 1
PALB2	partner and localizer of BRCA2
PAR	poly-ADP-ribose
PIN1	peptidyl-prolyl cis-trans isomerase NIMA-interacting 1
RAD51	DNA repair protein RAD51 homolog 1
RBBP8	RB binding protein-8
RNF168	RING-finger protein 168
RPA	replication protein A
ssDNA	single stranded DNA

## Footnotes

We apologize to those authors that we could not include due to reference restrictions.
